# Supplementary material for: Interaction of Potato Autophagy-Related StATG8 Family Proteins with Pathogen Effector and WRKY Transcription Factor in the Nucleus
Source: Microorganisms. 2025 Jul 5;13(7):1589. doi: 10.3390/microorganisms13071589 (PMC12299205; doi:10.3390/microorganisms13071589)
Supplement: Supplementary file 1 [file microorganisms-13-01589-s001.zip › 2025-Huh_StATG8-Supporting data F.pdf]

Supporting information (Figure S1-S6), Smovie1, and Tables (Table S1-S2)

Fig. S1. StATG8 proteins in plants are predominantly found in the cytoplasm, autophagosomes, and nucleus. The subcellular localization of GFP-StATG8-2.1 near the tip of transgenic roots was examined. In the intracellular location of GFP-StATG8-2.1 at the root tip, GFP signals were observed in complex patterns across multiple layers of cells.

Fig. S2. Rendering sequence similarity and secondary structure information of aligned sequences of human LC3 and StATG8 family proteins. The sequences of human LC3 (Q9H492) and StATG8 family proteins were aligned, and their secondary structures were subsequently analyzed using ESPript. This comparative analysis allows for the examination of similarities and differences between the amino acid sequences and secondary structures of these proteins. In the representation, the faint yellow box highlights the AIM, as predicted by the iLIR Autophagy Database. Additionally, the dotted box denotes the AIM, present at the same location in both human LC3 and potato StATG8. These motifs might play roles in the interactions and functions of the respective proteins in autophagy-related process.

Fig. S3. In the BiFC negative control test, mRYCE-StATG8 did not produce any BiFC signal when co-expressed with the empty mRYNE vector.

To test for potential false-positive BiFC signals, StATG8 family genes cloned into the mRYCE vector were transiently expressed in *N. benthamiana* leaves together with the empty mRYNE vector. At 3 days post-infiltration (dpi), BiFC signals were examined using confocal microscopy.

Fig. S4. The PopP2-mCherry fusion protein was predominantly localized in the nucleus, although a portion was also observed in the cytoplasm.

The *35S::PopP2-mCherry* construct was transiently expressed in *N. benthamiana* leaves, and fluorescence signals were observed using confocal microscopy at 3 days post-infiltration (dpi). PopP2-mCherry signals were mainly observed in the nucleus. Cytoplasmic localization is also evident, as indicated by arrows.

Fig. S5. PopP2 and StATG8-2.1 exhibited reconstituted mCherry signals in the nucleus and autophagosomes in the BiFC assay.

The mRYNE-PopP2/mRYCE-StATG8-2.1 construct or the negative control mRYNE-PopP2/mRYCE empty vector construct was transiently expressed in *N. benthamiana* leaves. Reconstituted BiFC mCherry signals were detected by confocal microscopy at 3 dpi. Arrows indicate autophagosomes.

Fig. S6. Reconstituted mCherry fluorescence signals were observed in the nucleus and autophagosomes in the BiFC assay with StATG8-2.1 and AtWRKY40.

The mRYNE-StATG8-2.1/mRYCE-AtWRKY40 construct, or the negative control mRYNE/mRYCE-AtWRKY40 construct, was transiently expressed in *N. benthamiana* leaves. Confocal microscopy performed at 3 dpi revealed BiFC-mediated mCherry and DAPI staining signals. Arrowheads indicate the presence of punctate structures corresponding to autophagosomes.

Smovie S1. Intracellular dynamics following transient expression of the fusion protein GFP-StATG8-2.1 in *N. benthamiana*

Table S1. BiFC and Y2H cloning primer list

Table S2. Transcription factors with putative AIM in Arabidopsis using the iLIR Autophagy Database

Supporting information Figure S1

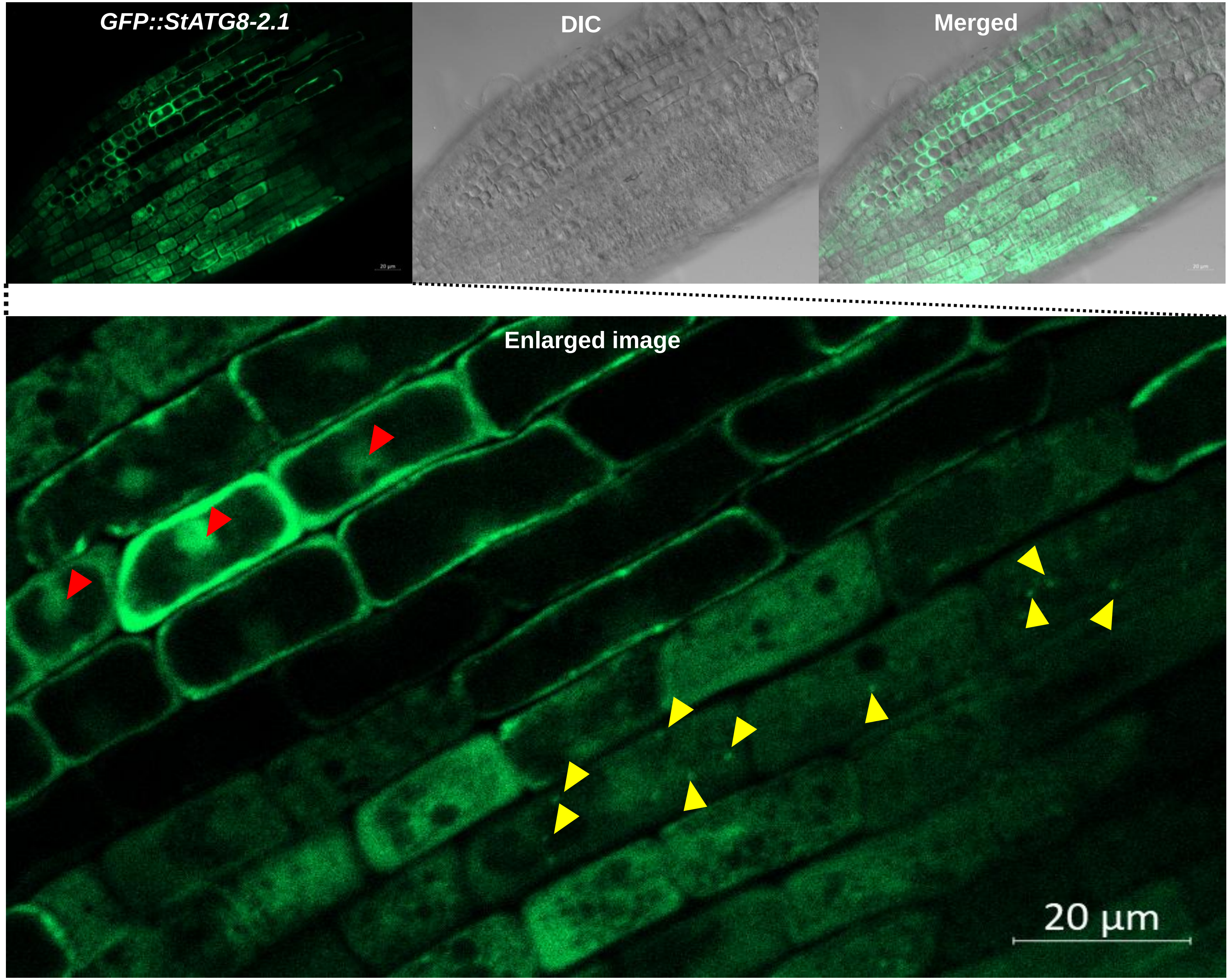

Supporting information Figure S2

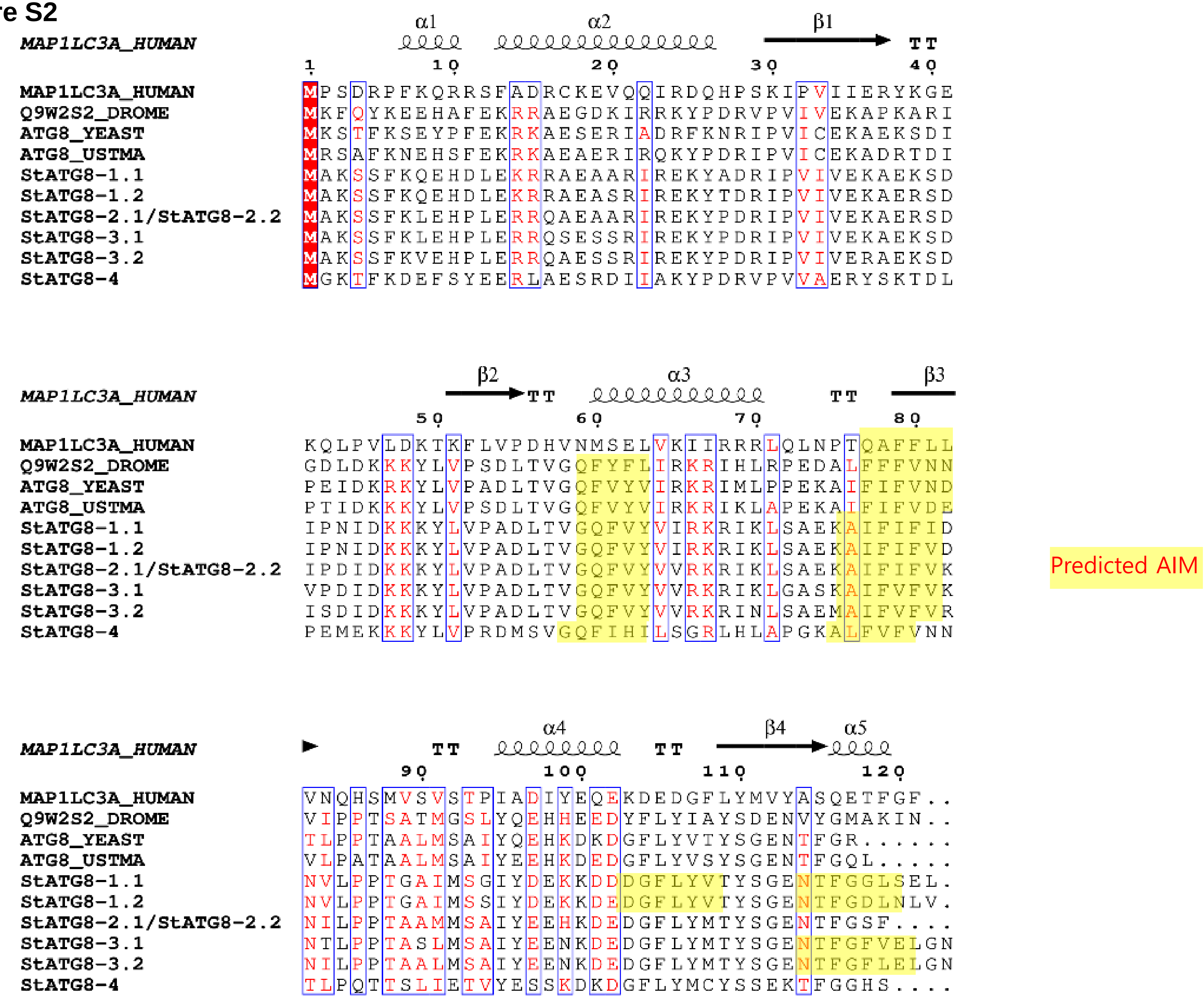

Supporting information Figure S3

Negative control

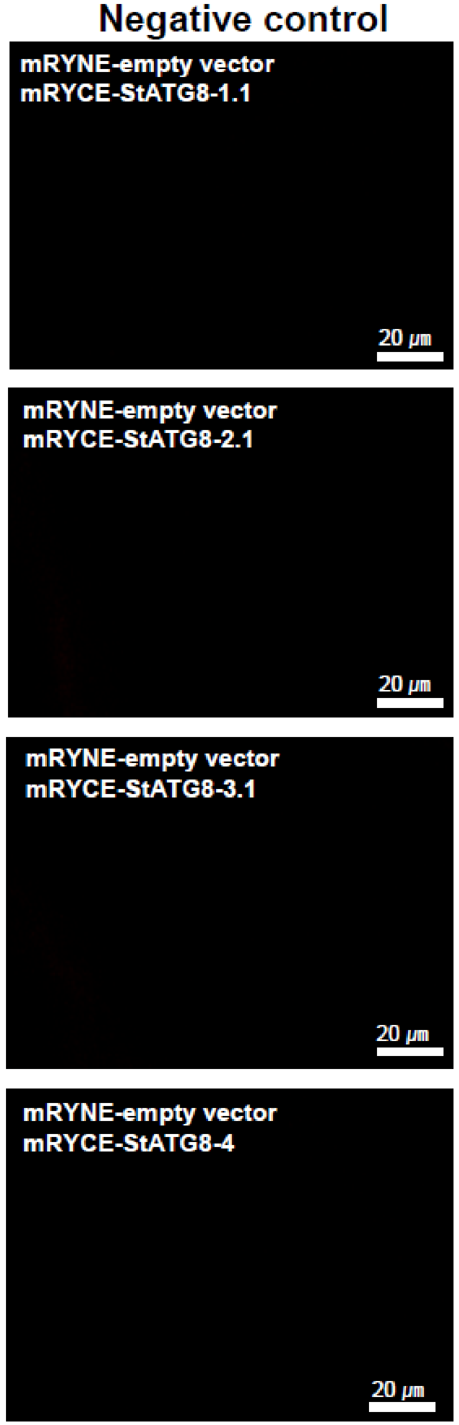

Supporting information Figure S4

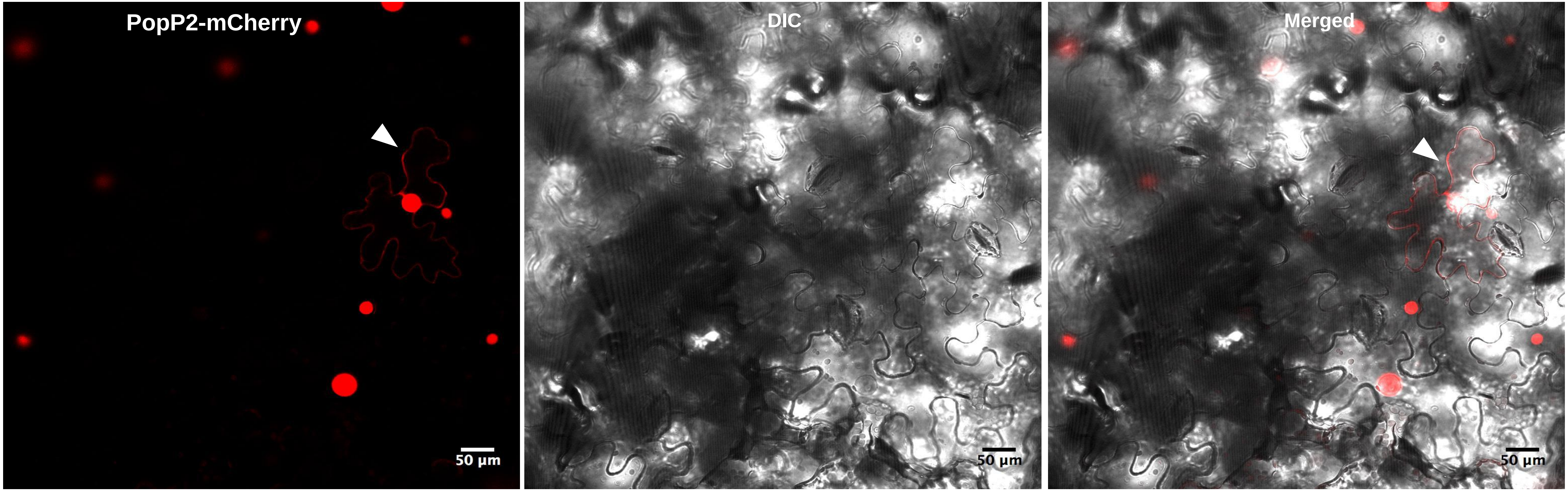

Supporting information Figure S5

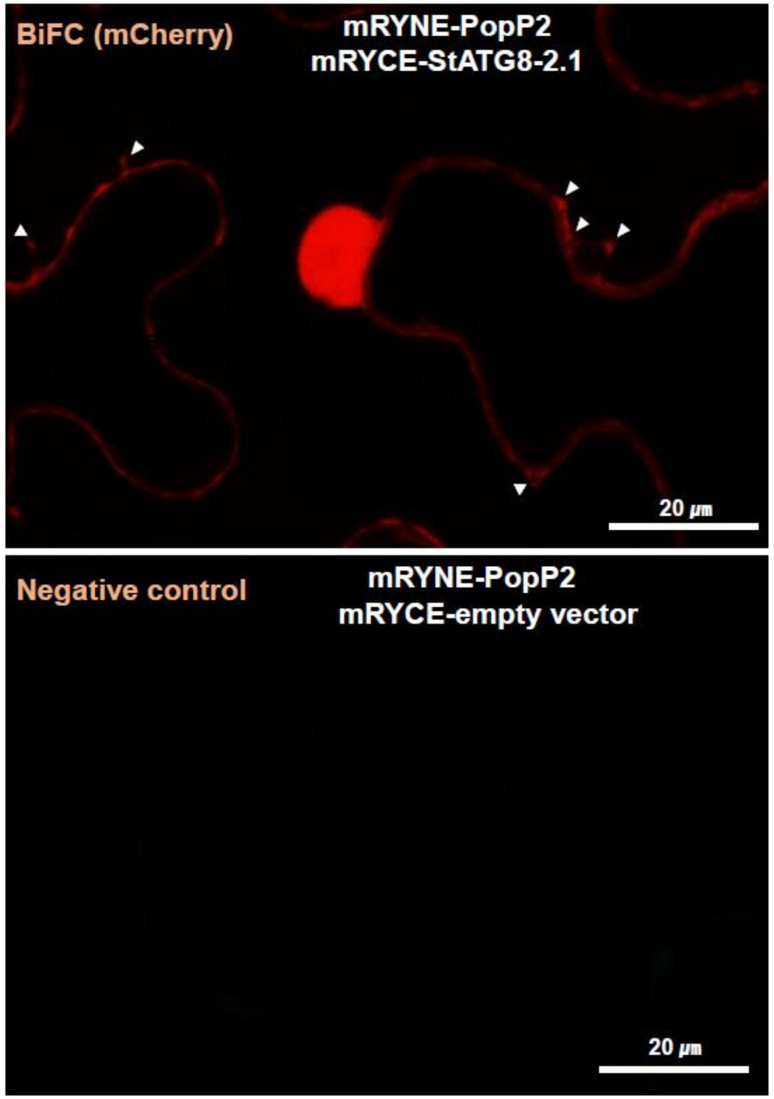

Supporting information Figure S6

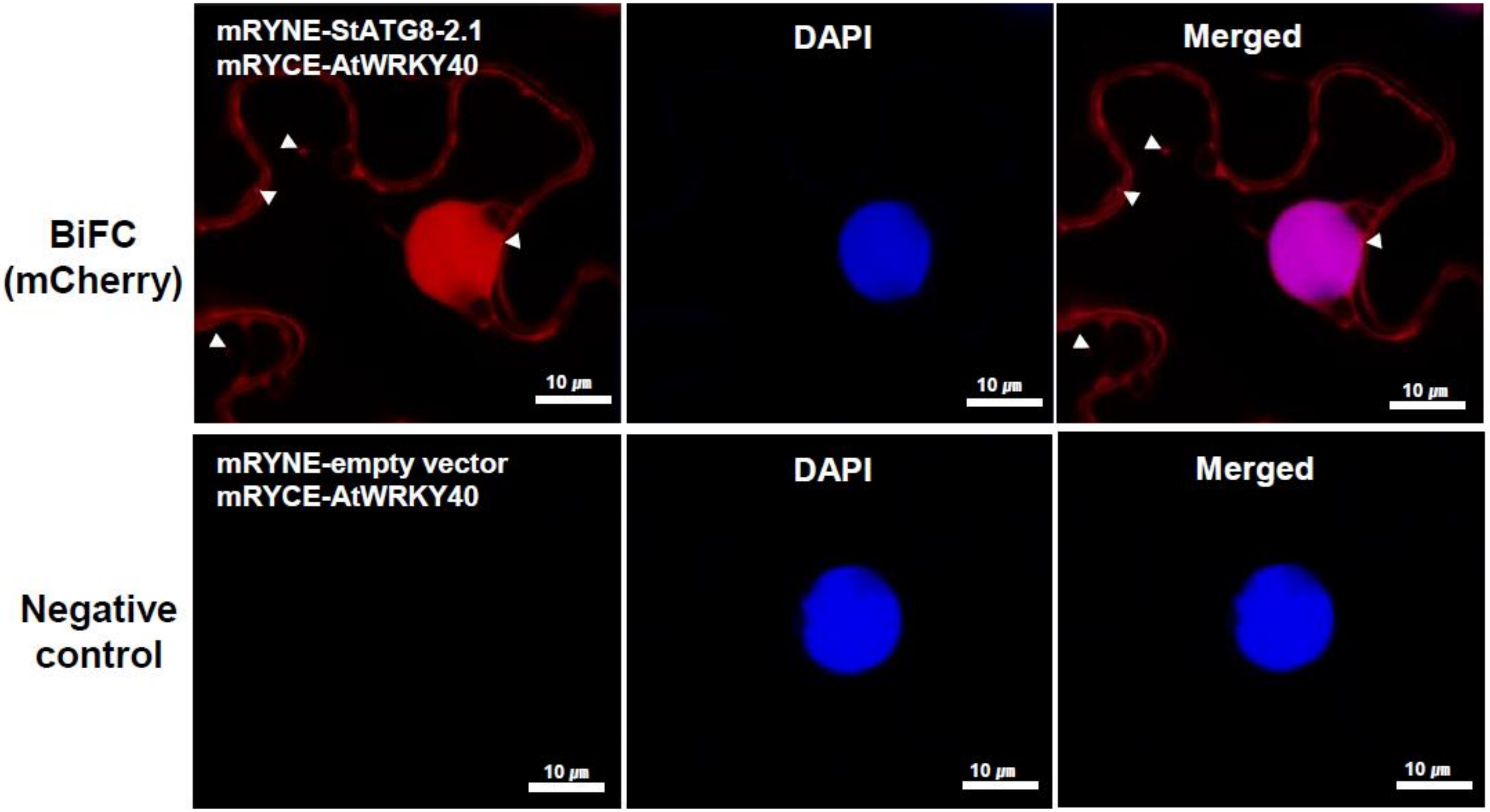

Table S1. BiFC and Y2H cloning primer lists

Primer name	Forward primer (5′ → 3′)	Reverse primer (5′ → 3′)
StATG8-1.1 for BiFC	ACTAGTATGGCTAAGAGCTCATTCAA	CCCGGGCAGTTCGCTCAGACCCCCGA
StATG8-1.2 for BiFC	ACTAGTATGGCAAAGAGTTCGTTCAA	CCCGGGCACTAAGTTAAGGTCCCCAA
StATG8-2.1 for BiFC	ACTAGTATGGCTAAAAGCTCCTTCAA	CCCGGGGAAGGATCCAAATGTATTCT
StATG8-2.2 for BiFC	ACTAGTATGGCCAAAAGCTCCTTCAA	CCCGGGAAAGGATCCGAAGGTATTCT
StATG8-3.1 for BiFC	ACTAGTATGGCCAAAAGTTCTTTCAA	CCCGGGATTTCCAAGCTCAACAAACC
StATG8-3.2 for BiFC	ACTAGTATGGCTAAGAGTTCTTTCAA	CCCGGGATTCCCAAGCTCGAGGAAAC
StATG8-4 for BiFC	ACTAGTATGGGGAAGACCTTCAAAGA	CCCGGGAGAGTGACCACCAAAGGTTT
PopP2/PopP2^{C321A} for BiFC	CTCGAGATGAAGGTCAGTAGCGCAAAC	CCCGGGGTTGGTATCCAATAGGGAATCC
AtWRKY18 for BiFC	CTCGAGATGGACGGTTCTTCGTTTCTC	CCCGGGTGTTCTAGATTGCTCCATTAACCTC
AtWRKY40 for BiFC	CTCGAGATGGATCAGTACTCATCCTCTTTG	CCCGGGTTTCTCGGTATGATTCTGTTGATAC
AtWRKY60 for BiFC	CTCGAGATGGACTATGATCCCAACACC	CCCGGGTGTTCTTGAATGCTCTATCAATCTCC
StATG8-1.1 for Y2H	GAATTCATGGCTAAGAGCTCATTC	GAATTCCTACAGTTCGCTCAGAC
StATG8-1.2 for Y2H	GAATTCATGGCTAAAAGCTCCTTC	GAATTCTCAGAAGGATCCAAATGTATTC
StATG8-3.1 for Y2H	CATATGATGGCCAAAAGTTCTTTC	CATATGTTAATTTCCAAGCTCAAC
StATG8-4 for Y2H	GAATTCATGGGGAAGACCTTCAAAG	GAATTCCTAAGAGTGACCACCAAAG
AtWRKY40 for Y2H	GAATTCATGGATCAGTACTCATCC	GAATTCCTATTTCTCGGTATGATTCTG
AtWRKY60 for Y2H	GAATTCATGGACTATGATCCCAAC	GAATTCTCATGTTCTTGAATGCTC

Table S2. Transcription factors with putative AIM in Arabidopsis

No.	Uniport ID	Motif	Protein information
*1	B3H7M2	FSFDSL	Basic-leucine zipper (BZIP) transcription factor family protein
*2	F4I6T7	FSFDDI	Transcription factor LHL3/LL1
*3	F4IGJ9	FSFDSL	Basic-leucine zipper (BZIP) transcription factor family protein
*4	F4IQH8	LDYIHV	Transcription factor bHLH64
*5	F4J9K3	AMFQLL	MADS-box transcription factor family protein
*6	F4J9K4	AMFQLL	MADS-box transcription factor family protein
*7	F4JGT7	ETFEEV	C2H2-type zinc finger transcription factor
*8	F4JN33	DEFVVV	NAC transcription factor-like 9
*9	F4JVA4	STYEDL	DNA binding / transcription factor
*10	F4JXW4	FDFQEL	Putative c-myb-like transcription factor MYB3R-4
*11	O04336	SGFQEL	Probable WRKY transcription factor 21
*12	O22208	SDFDSI	BZIP family transcription factor
*13	O22230	FVYTAL	Heat stress transcription factor B-3
*14	O22873	GGFDEL	BZIP transcription factor
*15	O23160	PTYLSL	MYB transcription factor
*16	O49515	FEFEDL	Ethylene-responsive transcription factor ERF091
*17	O49687	LSFTSV	Transcription factor MYC4
*18	O80340	PTFLEL	Ethylene-responsive transcription factor 4
*19	O81037	PSFLAL	Transcription factor bHLH70
*20	O81790	FSYDEI	NAC transcription factor-like 9
*21	O81821	PEFDLV	Heat stress transcription factor A-1b
*22	O82595	LVYDSV	B3 domain-containing transcription factor NGA4
*23	P42736	ESFLEL	Ethylene-responsive transcription factor RAP2-3
*24	Q01593	PEFLPL	B3 domain-containing transcription factor ABI3
*25	Q0JXE7	KDYIHV	Transcription factor BPE
*26	Q0JXE7-2	KDYIHV	Isoform 2 of Transcription factor BPE
*27	Q39117	SEFEIV	Trihelix transcription factor GT-2

*28	Q39162	EVYEPL	Transcription factor TGA4
*29	Q3EAI1	GSFTAL	Transcription factor bHLH60
*30	Q3EAI1-2	GSFTAL	Isoform 2 of Transcription factor bHLH60
*31	Q7XJU0	FSFDDI	Transcription factor bHLH157
*32	Q7XJU0-2	FSFDDI	Isoform 2 of Transcription factor bHLH157
*33	Q8GX46	FTYDPL	Transcription factor bHLH91
*34	Q8H1F0	FSFDSL	Basic-leucine zipper (BZIP) transcription factor family protein
*35	Q8L5Y2	KEFQSL	BZIP transcription factor-like protein
*36	Q8S3D2	PSFLPL	Transcription factor bHLH87
*37	Q8VWG0	SSFIDV	AtbZIP transcription factor
*38	Q8VWR2	SSFIDV	AtbZIP transcription factor
*39	Q8VWV6	KDFDIL	Probable WRKY transcription factor 61
*40	Q8VZQ2	FGFQKL	MYB transcription factor
*41	Q93VJ4	PDYIHV	Transcription factor BEE 2
*42	Q93VJ4-2	PDYIHV	Isoform 2 of Transcription factor BEE 2
*43	Q94FL9	FDFQEL	MYB transcription factor
*44	Q94ID6	PGYQVV	Ethylene-responsive transcription factor 12
*45	Q9C538	PSYQAL	C2H2 type zinc finger transcription factor-like protein
*46	Q9C5T4	SSFLDI	WRKY transcription factor 18
*47	Q9C882-3	GEYEKI	Isoform 3 of Trihelix transcription factor GTL1
*48	Q9C8P8	DSFEFL	Transcription factor bHLH80
*49	Q9CA27	GTFDTL	Ethylene-responsive transcription factor ERF118
*50	Q9CA27	LEFDAI	Ethylene-responsive transcription factor ERF118
*51	Q9CAA9	DGYIHV	Transcription factor bHLH49
*52	Q9CAA9-2	DGYIHV	Isoform 2 of Transcription factor bHLH49
*53	Q9FGP7	FTYDFL	Nuclear transcription factor Y subunit C-6
*54	Q9FH19	SSYQAL	C2H2 type zinc finger transcription factor-like protein
*55	Q9FH57	EEFLAV	GATA transcription factor 5
*56	Q9FJL4	KDYIHV	Transcription factor bHLH78
*57	Q9FJL4	RSFTHL	Transcription factor bHLH78

*58	Q9FLM6	KGFDIV	Transcription factor TCP6
*59	Q9FNY2	RVYDAL	Transcription factor-like protein DPB
*60	Q9FNY2-2	RVYDAL	Isoform 2 of Transcription factor-like protein DPB
*61	Q9FV71	ESFEDI	Transcription factor E2FB
*62	Q9FV71-2	ESFEDI	Isoform 2 of Transcription factor E2FB
*63	Q9FYJ6	RSFLDI	Transcription factor bHLH111
*64	Q9LG05	EVFDDI	Probable WRKY transcription factor 10
*65	Q9LK48	KDYIHV	Transcription factor bHLH77
*66	Q9LND0	GMFEEI	Transcription factor bHLH89
*67	Q9LQ28	ADYEEL	Ethylene-responsive transcription factor ERF022
*68	Q9LSD5	KDFQIV	Transcription factor TCP20
*69	Q9LT23	SEYTAL	Transcription factor bHLH121
*70	Q9LT23-2	SEYTAL	Isoform 2 of Transcription factor bHLH121
*71	Q9LT45	DEYVLI	GATA transcription factor 29
*72	Q9LTS4	ESFQAL	Putative transcription factor bHLH041
*73	Q9LUH8	KMYLTL	Heat stress transcription factor A-6b
*74	Q9LV17	KDYIHV	Transcription factor bHLH79
*75	Q9LXA9	KSFISI	Transcription factor bHLH61
*76	Q9M041	EGFLSV	Transcription factor bHLH140
*77	Q9M0J3	GSYVPV	Ethylene-responsive transcription factor ERF054
*78	Q9M322-2	DDYTHL	Isoform 2 of Probable RNA polymerase II transcription factor B subunit 1-3
*79	Q9M9L1	AMFQLL	MADS-box transcription factor family protein
*80	Q9SG82	PSFVPI	Myb family transcription factor
*81	Q9SG82	PSFLAV	Myb family transcription factor
*82	Q9SI37-2	DVFTAV	Isoform 2 of WRKY transcription factor 1
*83	Q9SIG8	FSFDSL	Basic-leucine zipper (BZIP) transcription factor family protein
*84	Q9SK03	LSFDIL	Ethylene-responsive transcription factor RAP2-7
*85	Q9SK03-2	LSFDIL	Isoform 2 of Ethylene-responsive transcription factor RAP2-7
*86	Q9SK55	ASWDEL	Transcription factor JUNGBRUNNEN 1
*87	Q9SRT2	KDYIHV	Transcription factor bHLH62

*88	Q9STX0	EEFLKL	Probable WRKY transcription factor 7
*89	Q9SZ67	STWDFV	Probable WRKY transcription factor 19
*90	Q9SZ67	AMFIPL	Probable WRKY transcription factor 19
*91	Q9T062	AGFVKI	BZIP transcription factor-like protein
*92	Q9T0D3	KTYQLV	Heat stress transcription factor B-2b
*93	Q9T0D3	GSWLEL	Heat stress transcription factor B-2b
*94	Q9T0J7	FSFDSL	Basic-leucine zipper (BZIP) transcription factor family protein
*95	Q9ZPW3	LDYIHV	Transcription factor HBI1
*96	Q9ZPW3-2	LDYIHV	Isoform 2 of Transcription factor HBI1
*97	Q9ZPZ8	ATFLSV	Myb family transcription factor
*98	Q9SAH7	DNYNVL	WRKY transcription factor 40
*99	Q9SK33	EKYYAL	WRKY transcription factor 60
